# Enzyme overexpression – an exercise toward understanding regulation of heparan sulfate biosynthesis

**DOI:** 10.1038/srep31242

**Published:** 2016-08-11

**Authors:** Jianping Fang, Tianyi Song, Ulf Lindahl, Jin-Ping Li

**Affiliations:** 1 Department of Medical Biochemistry and Microbiology, University of Uppsala, Uppsala, Sweden; 2 SciLifeLab, University of Uppsala, Uppsala, Sweden

## Abstract

Biosynthesis of heparan sulfate (HS) involves conversion of D-glucuronic acid (GlcA) to L-iduronic acid (IdoA) units catalyzed by glucuronyl C5-epimerase (Hsepi). IdoA units are the favored substrate for 2-O-sulfotransferase (2OST). We used HEK293 cells as a model to investigate the effects of overexpression of these enzymes on HS structure. Overexpression of Hsepi alone resulted in an unexpected increase in HS chain length. A Hsepi point-mutant (Y168A), devoid of catalytic activity, failed to affect chain length. Moreover, the effect of Hsepi overexpression on HS chain length was abolished by simultaneous overexpression of 2OST. These findings raise novel aspects on regulation of HS biosynthesis. We propose a hypothetical enzyme-binding protein (EBP) with distinct, specific and partly overlapping binding sites, the interactions of which will determine levels of enzymes available to the biosynthetic process.

Heparan sulfate (HS) is a glycosaminoglycan of complex and variable structure, ubiquitously expressed in proteoglycan form on cell surfaces and in the extracellular matrix. HS is essential in animal development and plays critical roles in homeostasis and various disease conditions. The diverse effects of HS are primarily due to non-covalent, mainly ionic, interactions with a multitude of proteins, including growth factors and their receptors, cytokines, enzymes and inhibitors, microbial proteins and proteins of the extracellular matrix^1^,^2^,^3^. The functional variability reflects the marked structural diversity of HS polymer, which appears to be strictly regulated in cell/tissue-specific manner^4^,^5^,^6^. HS biosynthesis is initiated by formation of a polymer composed of repeating [GlcAβ1,4-GlcNAcα1,4] disaccharide units. This precursor polysaccharide subsequently undergoes a series of modification reactions, including N-deacetylation/N-sulfation of GlcNAc residues, C5-epimerization of GlcA to IdoA units and O-sulfation at various positions of the hexuronic acid and glucosamine residues^1^,^4^. Whereas several of the corresponding enzymes show multiple isoforms encoded by distinct genes, the glucuronyl C5-epimerase (Hsepi) and hexuronyl 2-O-sulfotransferase (2OST) are encoded by single genes, *Glce* and *Hs2st*, respectively^7^,^8^. The modifications occur in essentially sequential order, such that the products of one reaction form the substrate for the next; yet each step is incomplete in the sense that only a fraction of the available substrate sites is utilized. Thus the initial modification, N-deacetylation and N-sulfation of GlcNAc residues, yields domains of N-acetylated or N-sulfated sequences, where subsequent reactions (C5-epimerization, O-sulfations) are largely restricted to the latter domains^1^. The structural diversity of the final products reflects the variable extent of the different modification steps.

The mechanism behind the regulation of the overall modification process remains, by and large, a mystery. The ‘GAGosome’ concept, while still conjectural, features a tightly knit complex of enzymes interacting with each other, their substrates, and, potentially, additional auxiliary proteins^4^,^9^,^10^. Regulation of HS biosynthesis may presumably be understood in terms of GAGosome organization and mode of action. Whereas the GAGosome has so far evaded direct analysis by available techniques, indirect approaches have involved genetic manipulation of the various enzymes concerned followed by analysis of resulting HS structures. In the present study, we have examined the effect of Hsepi overexpression in HEK293 cells. Unexpectedly, Hsepi overexpression led to increase in HS chain length, which was reverted by co-overexpression of 2OST. Possible mechanisms behind these findings are discussed, based on a notion of enzyme regulation through protein/protein interactions.

## Results

### Overexpression of glucuronyl C5-epimerase increases HS chain length in HEK293 cells

**Figure 1:**
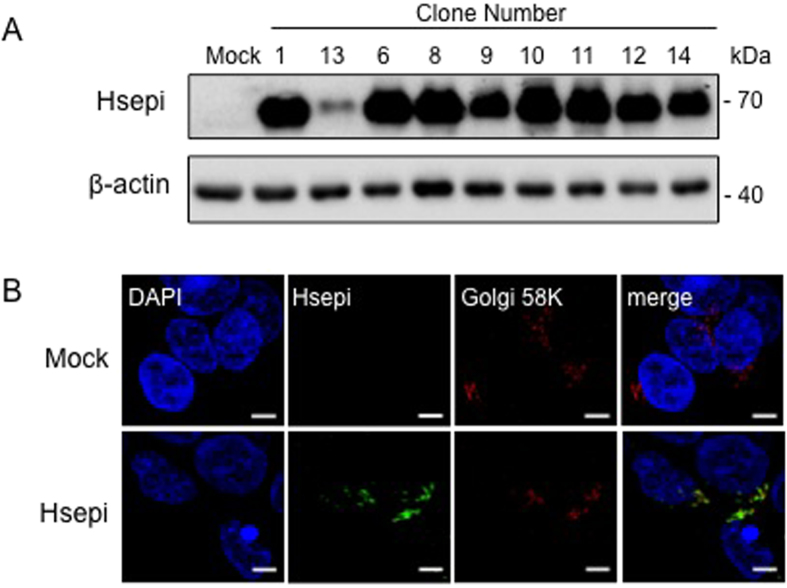
Overexpression of Hsepi in HEK293 cells. HEK293 cells were transfected with a lentivirus expression plasmid carrying the human Hsepi gene (*Glce*). (**A**) Western blot analysis of the cell lysate (40 μg protein) with an antibody against Hsepi showed overexpression of Hsepi in the stably transfected cells. The endogenous Hsepi in Mock transfected cells was below the detection limit. (**B**) Immunocytochemical staining with antibodies against Hsepi (*green*) and a Golgi 58K protein (*red*) showed the Golgi localization of Hsepi. Scale bars indicate 5 μm in the images.

Full-length recombinant human Hsepi was stably expressed in HEK293 cells using a lentivirus system. Overexpression in several cell clones was confirmed by Western blot analysis with an anti-Hsepi antibody, that revealed strong 70 kDa bands corresponding to full-size Hsepi (617 aa) (Fig. 1A). The band was relatively weaker in Clone 13 (which, nevertheless, showed appreciable catalytic activity), and was undetectable in the mock-transfected clone indicating low endogenous expression level. Golgi localization of the overexpressed protein was confirmed by immunostaining of the cells (Fig. 1B).

**Figure 2:**
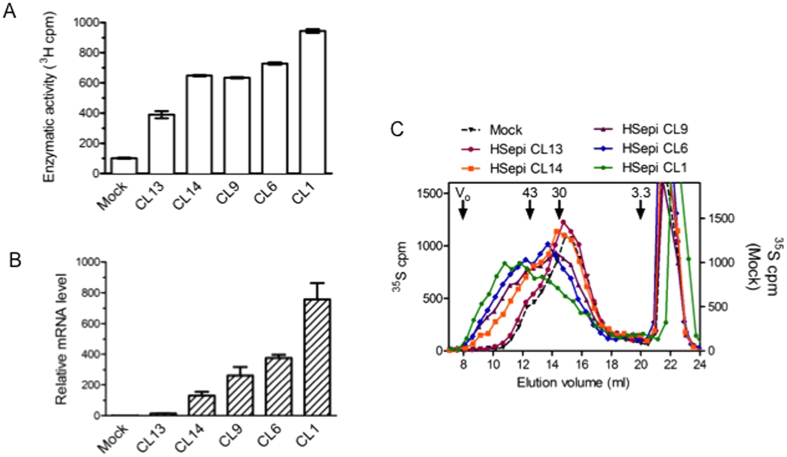
Chain length of HS in HEK293 cells expressing Hsepi at different levels. (**A**) Quantification of ^3^H release upon incubation of cell lysate with the substrate demonstrates the highest level of epimerase activity in clone 1. The data are presented as the mean ± SEM of triplicate analyses from two or three independent experiments; (**B**) Q-RT-PCR analysis shows corresponding levels of *Glce* mRNA in the transfected cells; (**C**) Gel chromatography of metabolically ^35^S-labeled HS on a Superose-6 column shows increased chain length of HS produced in the Hsepi-transfected clones, correlated with the level of Hsepi expression. Elution positions of polysaccharide standards (43, 30 and 3.3 kDa, respectively), are indicated^11^. The peaks eluted after 20 ml are degradation products of chondroitin sulfates.

Analysis of cell lysates showed increased epimerase activity in all clones investigated (Fig. 2A), in accord with the increased mRNA expression levels (Fig. 2B). However, there was no strict correlation, as illustrated in particular by Clone 13 that showed a relatively faint band on western blotting and a low level of mRNA expression, yet appreciable catalytic epimerase activity. Metabolic ^35^S-labeling of HS revealed an unexpected increase in chain length that correlated with Hsepi expression levels (Fig. 2C). HS chains isolated from Clone 1, with the highest levels of mRNA expression as well as Hsepi catalytic activity, thus showed an elution profile on Superose-6 gel chromatography that was markedly shifted compared to elution pattern of HS from mock-transfected cells. Notably, HS chains produced by Clone 13, containing low amounts of enzyme protein, did not differ from those of mock-transfected control cells. Estimates based on previous calibration with polysaccharide standards^11^ point to peak-elution components of >50 kDa and <30 kDa in Clone 1 and mock samples, respectively. Labeled HS samples from other clones emerged at intermediate positions correlating with Hsepi expression levels (Fig. 2B).

### Variable HS chain length is not due to heparanase action

**Figure 3:**
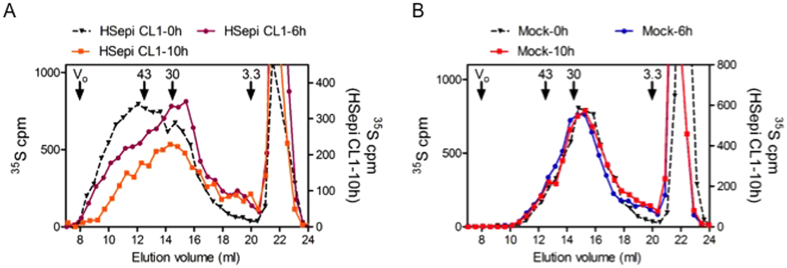
Pulse-chase labeling of HS in HEK293 cells. Gel chromatography on Superose-6 of HS samples isolated from Hsepi Clone 1 (**A**) and Mock cells (**B**) after metabolic ^35^S pulse-labeling for 30 min followed by chase-incubation for the indicated periods of time. Elution positions of polysaccharide standards (43, 30 and 3.3 kDa, respectively), are indicated. The peaks eluted after 20 ml are degradation products of chondroitin sulfates.

Mammalian cells express heparanase, an endo-ß-glucuronidase cleaving HS chains at internally located ß-glucuronidic linkages^12^. It was therefore important to assess whether the exceedingly long HS chains in Hsepi-overexpressing cells could be due to inhibition of the heparanase action. HS chains isolated after pulse ^35^S-labeling of Clone 1 for 30 min were again of high molecular weight, and decreased somewhat during subsequent chase incubation for up to 10 hr (Fig. 3A). By contrast, pulse-labeled HS chains from Mock cells were shorter from start and remained essentially unaffected throughout chase incubation (Fig. 3B). These findings establish that the discrepant chain lengths observed in Hsepi overexpression clones reflect effects on HS biosynthesis and not on degradation. Notably, transgenic overexpression of heparanase resulted in significant degradation of HS in Clone 1 as well as in the Mock clone (Suppl. Fig. 1A,B)

### Catalytically inactive C5-epimerase has no effect on HS chain length

**Figure 4:**
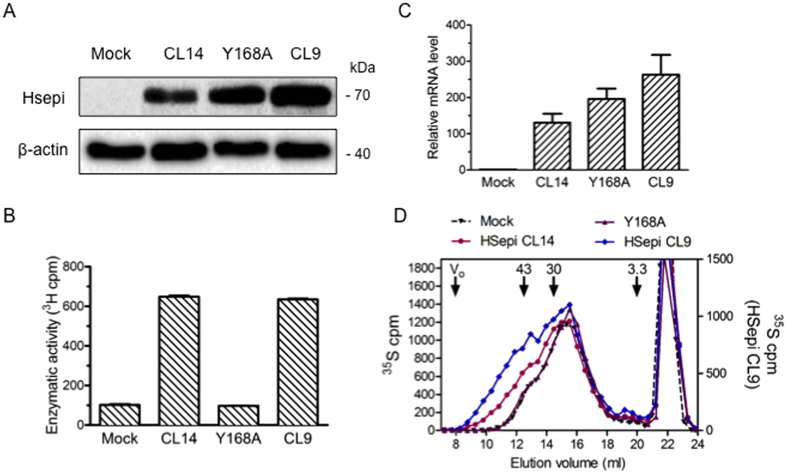
Characterization of HS from HEK293 cells expressing mutant Hsepi. (**A**) Western blot analysis of cell lysates (40 μg) shows overexpression of the mutant (Y168A point mutation) Hsepi, at a level comparable to that of Clone 9. (**B**) Epimerase activity of the mutant clone, assessed by ^3^H release upon incubation of cell lysates with labeled substrate, does not differ from that of the Mock clone. (**C**) Q-RT-PCR analysis confirms overexpression of *Glce* mRNA in the mutant clone. (**D**) Gel chromatography of ^35^S-labeled HS on a Superose-6 column shows no difference in chain length of HS isolated from Y168A and Mock clones.

We proceeded to investigate the dependence of HS chain elongation on Hsepi conformation. Was the excessive chain length due to nonspecific effects of accumulated Hsepi protein in the Golgi? To this end, a clone was prepared overexpressing a point-mutated (Y168A) Hsepi, previously shown to be devoid of catalytic activity^13^. While the mutant Hsepi expression was essentially equivalent to that of wildtype Clone 9 (Fig. 4A), the epimerase activity of mutant cell lysates was at the same level as that of mock-transfected cells (Fig. 4B). Further, whereas HS chains isolated from Clone 9 were extended as before (*cf.* Fig. 2C), HS chain length did not differ between the mutant Y168A and mock-transfected cells (Fig. 4C). Thus, the effect of Hsepi on HS chain length depends on retained catalytic epimerase activity.

### Effect of C5-epimerase overexpression on HS chain length is eliminated by simultaneous overexpression of 2-O-sulfotransferase

**Figure 5:**
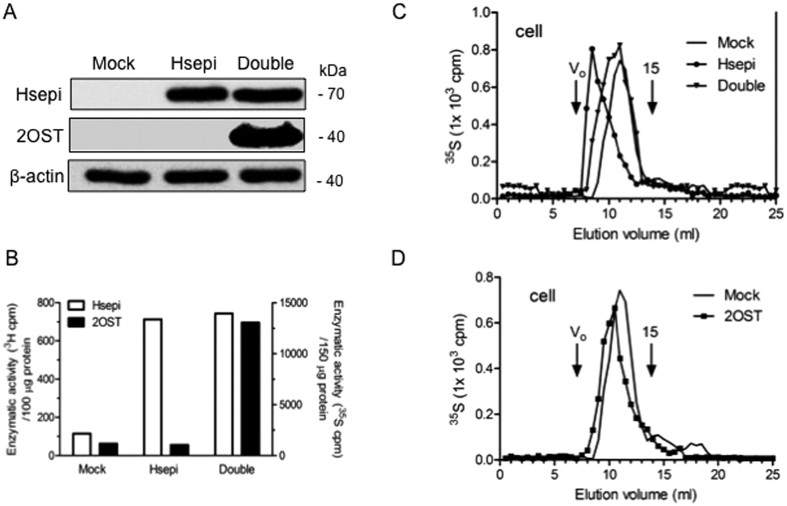
Double overexpression of 2OST and Hsepi restores wild-type HS chain length. (**A**) Western blot analysis of cell lysates (40 μg) stably overexpressing Hsepi and 2OST in HEK 293 cells. (**B**) Analysis of Hsepi (release of ^3^H) and 2OST (^35^S-incorporation) enzymatic activities in cell lysates. (**C,D**) Gel chromatography of metabolically ^35^S-labeled HS on a Superose-12 column. The peak elution position of a 15 kDa heparin marker is indicated. Before analysis, the chondroitin sulfate disaccharides generated by chondroitinase digestion were removed by DEAE-Sephacel chromatography.

IdoA residues generated by Hsepi action provide the preferred substrate for 2-O-sulfation^14^, and the two enzymes involved, Hsepi and 2OST, respectively, have been shown to interact in the cell^15^. It was therefore important to determine the effects, if any, of 2OST overexpression in cells previously transfected with Hsepi. We thus transfected the human *Hs2st* gene into Clone 1 cells, already stably overexpressing Hsepi, using the same lentivirus system. Western blot analysis confirmed overexpression of both proteins in the cell (Fig. 5A); moreover, the double-transfectant showed strongly increased 2OST activity compared to Mock- or Hsepi-alone-transfected cells (Fig. 5B). Immunocytostaining confirmed Golgi co-localization of the two proteins (Suppl. Fig. 2). Metabolically ^35^S-labeled HS was isolated from cell fraction of the double-transfected cells and analyzed by gel chromatography. HS produced by cells transfected with Hsepi alone again showed increased chain length compared to Mock polysaccharide (Fig. 5C). By contrast, HS chains generated by the double-transfected cells did not differ significantly from those of control HS. Overexpression of 2OST alone in the HEK293 cells did not appreciably affect HS chain length (Fig. 5D).

Notably, overexpression of *Glce* and/or *Hs2st* genes only marginally affected expression levels of other enzymes involved in HS biosynthesis (Suppl. Fig. 3).

## Discussion

Overexpression of Hsepi in HEK293 cells led to formation of longer HS chains compared to mock-transfected cells. This unexpected effect varied with and reflected levels of overexpression. It was important to assess whether the phenomenon was due to a specific mechanism rather than a nonspecific perturbation of the biosynthetic machinery, caused by excessive deposition of Hsepi protein in the Golgi (Fig. 1). Experimental findings strongly argue against the latter alternative. The effect of Hsepi overexpression on HS chain length thus was eliminated by simultaneous overexpression of 2OST (Fig. 5), in spite of apparent additional loading of the Golgi with enzyme proteins (Suppl. Fig. 2). Moreover, overexpression of the Y168A Hsepi mutant, devoid of epimerase activity, had essentially no effect on HS chain length (Fig. 4).

These findings suggest that a mechanism in control of chain elongation during HS biosynthesis is modulated by Hsepi, and that this modulation is somehow linked to catalytic activity of the protein. We cannot exclude a direct dependence on catalytic activity, involving formation of an IdoA residue in distinct position relative the non-reducing-terminal, growing end of the HS precursor chain. In fact, an effect akin to such a mechanism was previously attributed to N-sulfation of the penultimate glucosamine residue, catalyzed by a NDST enzyme^16^, and further supported by the finding of increased HS chain length resulting from NDST overexpression^17^. However, no corresponding critical IdoA residue has yet been identified. Moreover, the enzymes expressed by the different cell clones show variable proportions of catalytically active protein (Figs 1A and 2A,B), such that catalytic activity *per se* does not appear sufficient to affect HS chain length (see Clone 13; Fig. 2A,C). Instead, critical amounts of protein are needed, with a conformation compatible with (but not necessarily accompanied by) catalytic activity. Because of these considerations we favor a mechanism behind the effect of Hsepi on chain length based on protein-protein interactions.

Numerous reports have demonstrated interactions between enzymes catalyzing HS biosynthesis. The two exostosin proteins, EXT1 and EXT2, form a complex that expresses the combined GlcA- and GlcNAc-transferase activities required for generation of the HS precursor chain^18^,^19^. One of these, EXT2, was further shown to bind NDST1, such that EXT1 and EXT2 have opposing effects on Golgi expression of NDST1^10^. Importantly, Hsepi and 2OST interact during ER-to-Golgi transportation and, presumably, also once the Golgi destination has been reached^15^. Current notion predicts additional, yet undiscovered, interactions within the GAGosome framework (“modulators” in^1^). Here we consider the putative involvement of one such, still conjectural, enzyme binding protein (EBP), in control of HS chain elongation.

**Figure 6:**
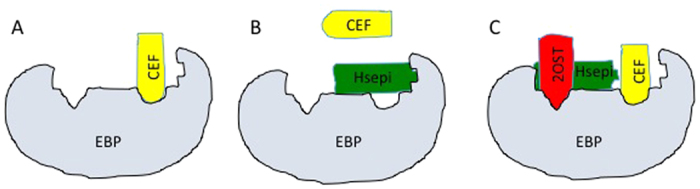
Speculative model of hypothetical Enzyme-Binding Protein (EBP) and its role in regulation of HS chain length. EBP features multiple, specific, partially overlapping binding sites for enzymes involved in HS biosynthesis. Regulation depends on availability of free enzymes, where EBP-bound enzymes are sequestered from participation in the biosynthetic process. (**A**) CEF (chain elongation factor; see the text) bound to EBP is unable to affect HS chain elongation. (**B**) Hsepi binds to EBP in two distinct modes, one of which dominates in the (relative) absence of 2OST and prevents simultaneous binding of CEF. CEF is free to promote HS chain elongation, as observed with cells overexpressing Hsepi alone. (**C**) Simultaneous overexpression of both Hsepi and 2OST generates Hsepi/2OST complex that binds to EBP such that the CEF-binding site is accessible. HS chains generated under these conditions will revert toward wild-type size.

The EBP, as illustrated in Fig. 6, exposes specific, partially overlapping enzyme-binding sites. Bound enzymes are sequestered from the biosynthesis process, contrary to unbound enzymes that are free to participate. EBP thus regulates the levels of available enzymes. The model illustrates one plausible mode of EBP action, which helps to explain, in particular, the effects of Hsepi overexpression on HS chain length (although other structural features are presumably modulated in related manner). The EBP is shown to bind a ”chain elongating factor” (CEF) that could be EXT1, overexpression of which results in formation of longer HS chains^20^, or an NDST enzyme responsible for incorporation of a critical, penultimate N-sulfate group^16^ (Fig. 6A). Hsepi binds to the EBP in two mutually excluding modes, one of which depends on complex formation with 2OST (Fig. 6B,C). Interaction with Hsepi alone, predominant after Hsepi overexpression, blocks binding of CEF to the EBP, thereby promoting chain elongation (Fig. 6B). By contrast, binding of the Hsepi/2OST complex will expose the binding site for CEF, which thus is sequestered and unavailable for chain elongation (Fig. 6C), in accord with the reversion of HS chain length to wild-type state (Fig. 5C).

The accumulated evidence, as noted above, implicating enzyme interactions in regulation of HS biosynthesis is compelling. Here we build on this concept in an attempt to explain the unexpected coupling between Hsepi overexpression and HS chain elongation. Other unexpected effects of genetic manipulation may reflect related or similar mechanisms. For instance, we note that reduced expression of EXTL3, another member of the exostosin member of glycosyltransferases, also results in increased HS chain length^21^. Importantly, studies using various animal model systems have shown that genetic manipulation of HS-modifying enzymes may influence activities of other enzymes catalyzing preceding steps in the overall biosynthesis process^22^,^23^. In particular, Hsepi in *C. elegans* was demonstrated to inhibit *N-*sulfation but stimulate both 2-*O-* and 6-*O-*sulfation^23^. Similarly, genetic ablation of the same enzyme in mice resulted in increased N-and 6-O-sulfation^24^. Clearly, the model proposed in Fig. 6 represents merely one of alternative scenarios to explain these findings. Others include, for instance, enzyme/protein interactions inducing conformational change of an EBP, resulting in modulation of enzyme availability. We hope that our model may provide a useful framework for future research on regulation of HS biosynthesis. Such studies would presumably benefit from further systematic experiments including co-immunoprecipitation using tagged enzyme and characterization of associated protein ligands.

## Methods

### Reagents

A polyclonal antibody against Hsepi was generated by commercial service (Innovagen, Sweden). A mouse monoclonal anti-2OST antibody was purchased from Santa Cruz, and the β-actin antibody used as an internal reference was from Abcam. A mouse anti-ERGIC53/p58 antibody was from Sigma. All Alexa Fluor conjugated antibodies were from Invitrogen. Substrate for Hsepi was prepared from metabolically C5-[^3^H]labeled K5-polysaccharide as described^25^.

### Overexpression of human genes in HEK293 cells

Lentivirus expression plasmids were generated by subcloning full-length human *Glce* from the cDNA ORF clone (Origene) into the XhoI/BamHI sites of a lentiviral vector carrying a puromycin resistance gene (pLVX-IRES-Puro). The full-length human *Hs2st* cDNA was subcloned onto the EcoRI/XbaI sites of a lentiviral vector carrying a neomycin resistance gene (pLVX-IRES-Neo). The identities of the expression plasmids were confirmed by sequencing (Eurofins). The single-site mutation of human *Glce* was prepared by PCR amplification using the construct of WT *Glce* in pLVX-IRES-Puro as template (Generay Biotechnology Company, Shanghai, China). Lentiviral particles carrying either the *Glce* or *Hs2st* gene were produced by transfection of HEK293T cells with the expression plasmids, psPAX2 packaging plasmid and the VSV-G-encoding plasmid pMD2.G. Media containing the lentivirus particles were collected and used to infect HEK293 cells. After infection, the cells were cultured in the presence of 3 μg/ml of puromycin (for Hsepi) or 1 mg/ml of G418 (for 2OST), respectively, for selection of clones. Cell pools stably expressing Hsepi or 2OST were diluted and monoclonal cell lines were obtained by sub-culturing the cells under the same antibiotic pressure. Selected clones stably expressing Hsepi were further transfected with 2OST, followed by selection with 1 mg/ml of G418 to obtain cell lines stably overexpressing both Hsepi and 2OST. Transient expression of heparanase was performed by transfection of pCDNA 3.1/Myc-His B plasmid carrying human heparanase gene (*Hspe*) as previously described^26^ and the vector pCDNA 3.1/Myc-His B was used as mock control.

### Determination of enzymatic activity

Hsepi activity was analyzed based on the release of ^3^H (as ^3^H_2_O) from a C5-^3^H-labeled polysaccharide substrate, essentially as described^27^. Cells at 90% confluence were lysed in homogenization buffer (50 mM HEPES pH 7.4, 100 mM KCl, 1% Triton X-100, 15 mM EDTA, containing protease inhibitors) and incubated on ice for 30 min. After centrifugation, total protein in the lysates was determined by the BCA assay (Thermo Scientific), and then mixed with the substrate (5,000 cpm). Following incubation at 37 °C for 20 min, released ^3^H_2_O was quantified by scintillation counting. The 2OST activity was determined as described^28^ using O-desulfated heparin as substrate and freshly prepared PAP^35^S as sulfate donor.

### Metabolic radiolabeling and isolation of HS from cells

The cells were cultured in DMEM supplemented with 10% FCS to 95% confluency. Following addition of 100 μCi/ml of Na^35^SO_4_ (specific activity 1500 Ci/mmol; Perkin Elmer), the cells were maintained in the same medium for 24 hr before harvesting. For isolation of HS, the medium was collected and the cells were washed with PBS and lysed in an extraction buffer containing 50 mM Tris-HCl pH 7.4, 4 M urea, 1% Triton X-100. After centrifugation, NaOH was added to the supernatants to a final concentration of 0.5 M and the samples were incubated on ice overnight to release HS chains from proteoglycan core proteins. After neutralization and centrifugation, the supernatants were diluted >10-fold with extraction buffer and applied to 2-ml DEAE-Sephacel columns (GE Healthcare Biosciences), equilibrated with extraction buffer. Following extensive washing of the columns with 50 mM NaAc pH 4.5, 0.25 M NaCl, bound material was eluted with 50 mM NaAc pH 4.5, 1.5 M NaCl. The eluted fractions were desalted in a PD-10 column and lyophilized. Products were treated with chondroitinase ABC (200 mU, Amsbio). In some experiments, the degraded chondroitin sulfate was removed by DEAE-Sephacel chromatography. Purity of HS was confirmed by treatment with HNO_2_ at pH 1.5 followed by gel chromatography.

For pulse-chase labeling, the cells were cultured in the presence of Na^35^SO_4_ (as above) for 30 min, and then washed with PBS, followed by chase-incubation with fresh medium lacking Na^35^SO_4_ for the indicated periods of time. HS was isolated as described above.

HS chain length was analyzed by gel chromatography on a Superose-6 or Superose-12 column (GE Healthcare Biosciences), eluted with 50 mM Hepes buffer, pH7.4 containing 1 M NaCl connected to a HPLC^29^. Peak elution volumes were assessed using size-defined glycosaminoglycans as previously described^11^ and indicated in the chromatograms.

### Western blot analysis

Cells were lysed with RIPA buffer (50 mM Tris, pH 7.5, 150 mM NaCl, 1% Triton X-100, 1% sodium deoxycholate, 1 mM EDTA, 0.1% SDS) for 30 min on ice. After centrifugation, supernatants were collected and protein concentration was determined by BCA assay. After denaturation, the samples (40 μg of total protein) were subjected to SDS-PAGE separation and blotting to nitrocellulose membranes that were incubated with the respective antibodies. Signals in the blots were developed with SuperSignal Pico or West Dura Substrate (Thermo Scientific) according to the manufacturer’s instructions.

### Immunocytochemistry

Cells grown on poly-L-lysine-coated coverslips were rinsed with PBS, fixed for 15 min with 4% PFA in PBS, and blocked with 0.3% Triton X-100 in PBS (PBST) containing 3% BSA. The cells were then incubated with primary antibodies (rabbit anti-Hsepi, mouse anti-p58, mouse anti-2OST) diluted 1:100 in PBST containing 3% BSA in a humid chamber at 4 °C overnight. After three washes, the cells were incubated with secondary antibodies (anti-rabbit IgG coupled with Alexa 488, or anti-mouse IgG coupled with Alexa 594 from Invitrogen) diluted 1:200 in PBST containing 3% BSA for 1 hr at RT. After repeated washing with PBS, coverslips were mounted with Vectashield mounting medium containing 4, 6-diamidino-2-phenylindole (DAPI) for nuclear staining (Vector Laboratories). Digital images were captured with a Zeiss LSM 700 confocal scanning microscope.

### Quantitative real-time (RT) PCR

Total RNA was isolated from the cells with QIAzol (Qiagen) following manufacturer’s instructions. The RNA (1–2 μg) was reversely transcribed to cDNA with Superscript II reverse transcriptase (Invitrogen) and diluted to a final volume of 100 μl. A 20 μl reaction mixture containing 2 μl of cDNA template, 0.4 μM primers and 10 μL SsoFast EvaGreen supermix (Bio-Rad) was added to a 48-well clear plate. Real-time PCR was performed using a MiniOpticon Real-Time PCR system with the Bio-Rad CFX manager software version 1.5. Conditions for amplification were 95 °C for 30 s and 40 cycles of 95 °C for 5 s followed by 60 °C for 10 s. The fold change of mRNA was evaluated by the relative copy number (RCN) and expression ratios of targeted genes normalized to the expression of the reference gene (housekeeping gene GAPDH). Ratios were calculated by the relative quantification method using the CFX manager software with the equation RCN = 2^−ΔΔCt^, where ΔC_t_ = Ct_target_ − Ct_reference_, and ΔΔCt = ΔCt_test sample_ − ΔCt_control sample_. The sequences of primers used are described in Table IV.

## Additional Information

**How to cite this article**: Fang, J. *et al*. Enzyme overexpression – an exercise toward understanding regulation of heparan sulfate biosynthesis. *Sci. Rep.* **6**, 31242; doi: 10.1038/srep31242 (2016).

## Supplementary Material

Supplementary Information
