# Supplementary material for: Enzyme overexpression – an exercise toward understanding regulation of heparan sulfate biosynthesis
Source: Sci Rep. 2016 Aug 11;6:31242. doi: 10.1038/srep31242 (PMC4980632; doi:10.1038/srep31242)
Supplement: Supplementary Information [file srep31242-s1.pdf]

Supplementary information

Enzyme overexpression – an exercise toward understanding regulation of heparan sulfate biosynthesis

Jianping Fang*, Tianyi Song, Ulf Lindahl and Jin-Ping Li*[1]

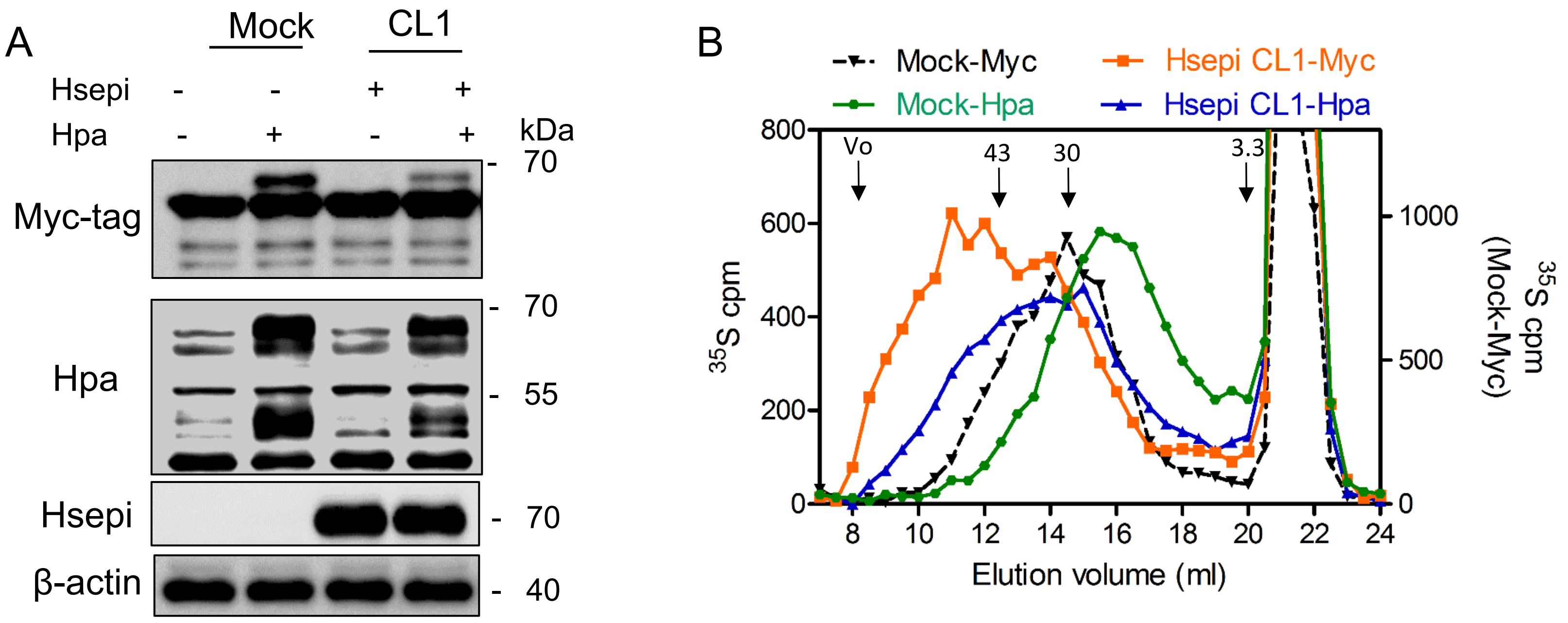

Suppl. Figure 1. *Overexpression of heparanase in HEK293 cells.* Mock and Clone 1 HEK293 cells were transiently transfected with a Myc-tagged expression plasmid carrying the human heparanase gene (*Hspe*). **A.** Western blot analysis of the cell lysate (40 µg protein) with antibodies against Myc, heparanase (Hpa), Hsepi and beta-actin. Blots show expression of both the 65 kDa (latent) and 50 kDa (active) forms of heparanase in both Mock and Hsepi Clone 1. **B.** Analysis of metabolically ^{35}S-labeled HS on a Superose-6 column shows that overexpression of heparanase led to reduction of HS chain length in both cell clones.

Fang et al, supplementary Fig.1

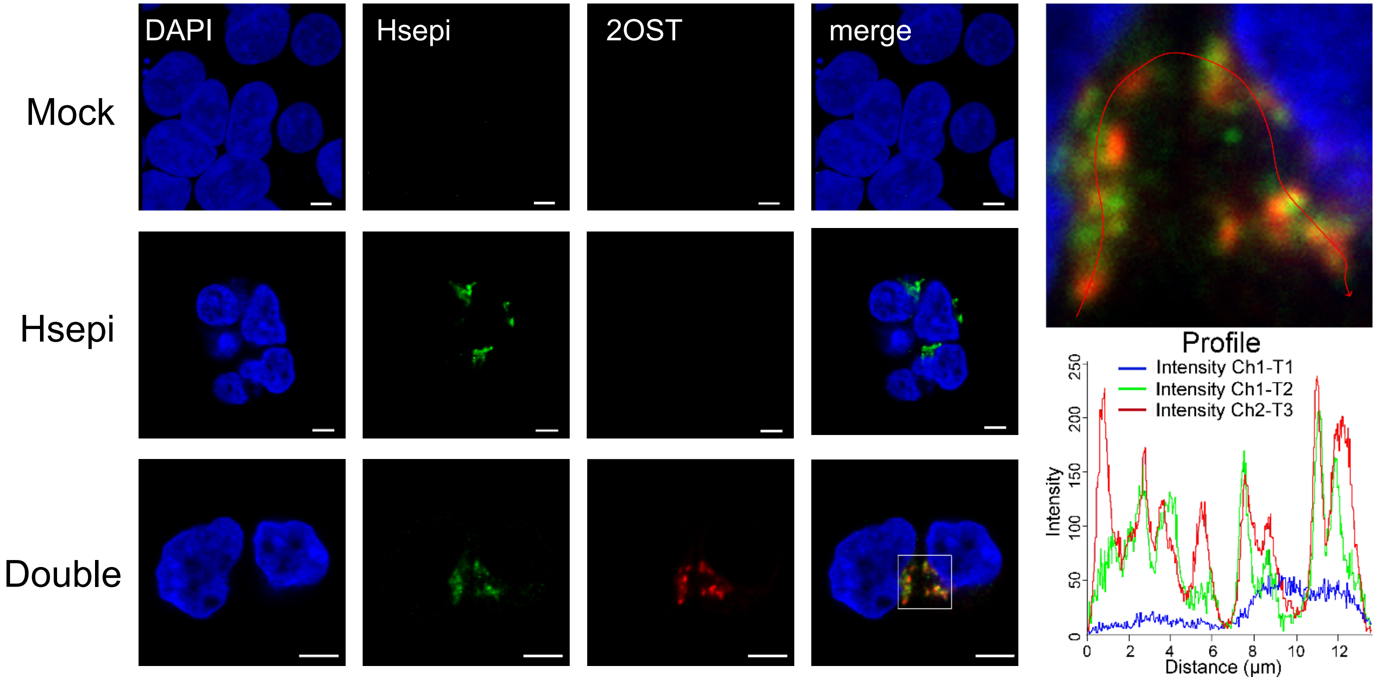

Suppl. Figure 2. Immunocytochemical staining of HEK293 cells showing colocalization of overexpressed Hsepi (*green*) and 2OST (*red*) in the Golgi. The profile of a confocal microscopy line scan from the enlarged image (top right panel) is shown in the right lower panel. Overlapped green (Hsepi) and red (2OST) lines indicate colocalization of Hsepi with 2OST. The blue line indicates scan of DAPI staining. Scale bars indicate 5 μm in the images.

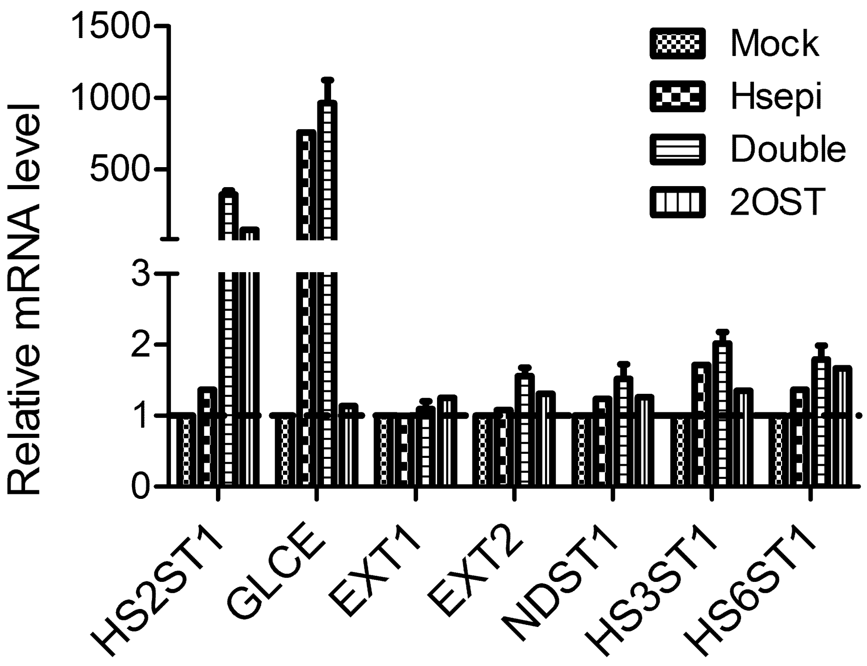

Suppl. Figure 3. *Gene expression in HEK293 cells stably overexpressing Glce and Hs2st .* Total RNA extracted from the cells, as indicated in the figure, was analyzed by qRT-PCR for the expression of genes encoding enzymes involved in HS biosynthesis (Ext1, Ext2, GlcA/GlcNAc co-polymerases; Ndst1, N-deacetylase/sulfotransferase 1; Hs3st1, GlcN 3-O-sulfotransferase 1; Hs6st1, GlcN 6-O-sulfotransferase 1). The copy number of the Mock-transfected sample is given as 1 (indicated by horizontal dotted line). Values represent the mean±SEM of triplicates from three independent experiments.
